# Engineering primary metabolism for sustainable production of isoprene in *Synechocystis* sp. PCC 6803

**DOI:** 10.1186/s12934-026-03040-8

**Published:** 2026-06-05

**Authors:** Paul Bolay, Kim N. Janssen, Stefan Timm, Martin Hagemann, Pia Lindberg

**Affiliations:** 1 Department of Chemistry - Ångström Laboratory, Uppsala University, Uppsala, 75120 Sweden; 2 Department Plant Physiology, Institute of Biosciences, University of Rostock, 18059 Rostock, Germany

**Keywords:** Cyanobacteria, Isoprene, Carbon partitioning, Nitrogen use efficiency, Metabolic engineering

## Abstract

The imminent climate crisis calls for the development of truly sustainable alternatives in virtually every aspect of human life. The photosynthetic production of chemicals like isoprene, a critical precursor for wide ranging applications, presents a promising alternative to conventional petroleum-based processes. While cyanobacteria can offer the ability for direct CO_2_ to isoprene conversion, highly efficient strains are required to reduce costs and resource usage. In this study, we enhanced isoprene productivity in the model cyanobacterium *Synechocystis* sp. PCC 6803 by engineering the primary metabolism of an isoprene producing strain. We inactivated synthesis of the carbon and energy storage polymer polyhydroxybutyrate (PHB) and increased isoprene synthase copy number in a previously published deletion mutant of PirC (*sll0994*), a recently discovered regulator involved in the fate of newly fixed carbon during limited nutrient supply. The engineered strains achieved an isoprene production of 278 µg*L^− 1^*OD^− 1^*h^− 1^ and showed elevated productivity under fluctuating nitrogen supply, ultimately resulting in significantly enhanced nitrogen use efficiency for isoprene production. As nitrate represents the ingredient with the highest cost contribution and climate impact in conventionally used cyanobacterial growth media, the presented strategy could reduce cost and environmental footprint without impeding performance for a wide array of compounds sustainably produced with cyanobacteria.

**Supplementary Information:**

The online version contains supplementary material available at 10.1186/s12934-026-03040-8.

## Background

Sustainable chemical production by photosynthetic microorganisms is an integral solution for developing a carbon-negative, zero waste circular economy necessary for minimizing human impact on climate change, resource scarcity, and pollution [1]. Isoprene is currently generated from crude oil sources with a global market size of 700 thousand tonnes per year expected to increase to 1200 thousand tonnes until 2032 [2]. Isoprene is used in large quantities for rubber production in the automotive industry, but can also serve as platform chemical or as jet fuel (upon direct photochemical polymerization by sunlight and hydrogenation), fulfilling the requirements for Grade A drop-in jet fuel [3] considerably enlarging future market size estimations. We have recently designed highly efficient isoprene producing *Synechocystis* sp. PCC 6803 (hereafter *Synechocystis*) strains that demonstrated unprecedented genetic and operational stability for photoautotrophic chassis, achieving long-term production in gram scale [4]. Due to its volatility and low boiling point, isoprene production can be performed in a continuous manner as it is constantly released from the culture, minimizing challenges in down-stream processing. Thus, isoprene represents a highly promising candidate for large-scale, sustainable *de novo* synthesis from CO_2_ in photosynthetic cyanobacteria. Moreover, its native role in mitigating heat and oxidative stress in plants, which is also observed in cyanobacteria engineered to produce isoprene, renders it a less burdensome or even beneficial target compound for the host [4, 5].

**Fig. 1:**
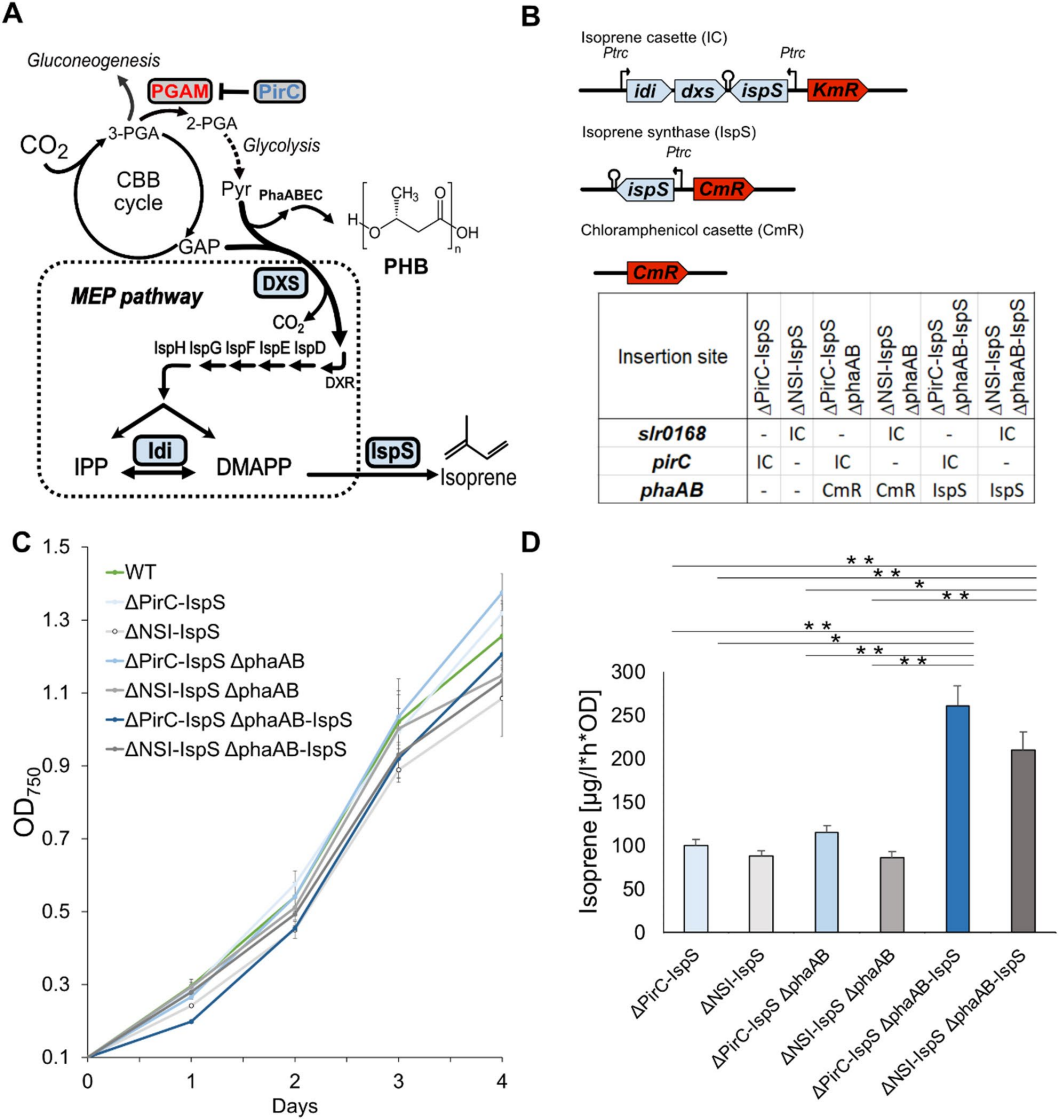
Elimination of PHB synthesis and introduction of second isoprene synthase copy **A** Metabolic scheme of isoprene production in *Synechocystis*. CBB cycle: Calvin-Benson-Bassham cycle, PGAM: Phosphoglycerate mutase, PirC: PII-interacting regulator of carbon metabolism, 3-PGA: 3-Phosphoglyceric acid, 2-PGA: 2-Phosphoglyceric acid, Pyr: Pyruvate, GAP: Glyceraldehyde-3-Phosphate, DXS: 1-deoxy-D-xylulose-5-phosphate synthase, DXR: 1-deoxy-D-xylulose-5-phosphate reductoisomerase, IspD: 2-C-Methyl-D-erythritol 4-phosphate-cytidylyltransferase, IspE: 4-diphosphocytidyl-2-C-methyl-D-erythritol kinase, IspF: 2-C-methyl-D-erythritol 2,4-cyclodiphosphate synthase, IspG: 4-Hydroxy-3-methyl-but-2-enyl pyrophosphate synthase, IspH: 4-Hydroxy-3-methyl-but-2-enyl pyrophosphate reductase, IDI: Isopentenylpyrophosphate isomerase, IspS: Isoprene synthase, IPP: Isopentenyl diphosphate, DMAPP: dimethylallyl diphosphate, PHB: Polyhydroxybutyrate **B** Top: Genetic constructs for *phaAB* deletion and introduction of second *ispS* copy under *Ptrc* control. Ptrc: Hybrid promoter consisting of -35 trp promoter and − 10 lacUV5 elements **B** Bottom: Overview of strains in this study **C** Growth comparison of generated strains in BG11 with 50 mM bicarbonate supplementation. **D** Isoprene formation on day 4. Data shown are average values and standard deviations from three independent replicates. Significance was tested using paired two samples Student’s *t*-test (ns = *P* > 0.05, * = *P* ≤ 0.05, ** = *P* ≤ 0.01)

One key limitation preventing competitiveness of chemicals production in photoautotrophic microorganisms is high production costs owed to the low volumetric productivities in comparison to hitherto developed heterotrophic processes [6]. To tackle this issue, we sought to enhance isoprene productivity by engineering regulation of carbon (C) metabolism towards elevated flux into precursors of isoprene synthesis. Intriguingly, cyanobacteria such as *Synechocystis* feature regulatory mechanisms that are absent in other bacterial clades, likely owing to their photoautotrophic lifestyle, leading to strong metabolic plasticity which counteracts engineering efforts that aim at streamlining metabolism for over-production [7]. One such feature is the use of small regulatory proteins for central metabolic processes, governed by the PII protein, a ubiquitous signal transducer key for metabolic regulation [8–10]. Here, we selected the recently characterized small regulatory protein PirC (PII interacting regulator of carbon metabolism) encoded by the *sll0944* gene [9, 11] for regulatory engineering. During nitrogen (N) starvation, PirC fulfills a crucial function in primary metabolism of *Synechocystis* by inhibiting its regulatory target phosphoglycerate mutase (henceforth PGAM) via protein-protein interaction. This is governed in a 2-oxoglutarate (2-OG) dependent manner via the signal transducing protein PII [9]. As 2-OG acts as the C skeleton for N assimilation via the GS-GOGAT cycle, its abundance is inversely correlated with N availability and thus perfectly serves as a proxy for sensing the C/N state of the cell [12]. PGAM, the target of PirC is a crucial switch for C partitioning in cyanobacteria as its activity determines whether newly assimilated C from the Calvin-Benson-Bassham (CBB) cycle is directed towards lower glycolysis or glycogenesis (Fig. 1A). Reduced PGAM activity results in an increased level of 3-phosphoglycerate (3-PGA). Subsequently, glycogen synthesis is induced by 3-PGA accumulation and its conversion via glyceraldehyde-3-phosphate (GAP) into gluconeogenesis under prolonged N starvation [13]. Furthermore, glycogen turnover in N starved cells results in accumulation of the energy and storage polymer polyhydroxybutyrate (PHB) in later phases of N starvation [14]. PHB is synthesized in an NADPH-consuming, multi-step process from acetyl-CoA, which is encoded by the *phaAB* and *phaEC* genes organized in two operons (in *Synechocystis*) that are upregulated during N starvation [15]. Upon prolonged N starvation, *pirC* deletion mutants accumulate higher levels of pyruvate than WT [9]. As the substrates for isoprene formation in *Synechocystis* are provided by the methylerythritol phosphate (MEP) pathway, that is fed by pyruvate and the CBB cycle intermediate GAP, *pirC* deletion could be beneficial for re-directing carbon and energy toward isoprene formation.

Few attempts have been made to utilize PirC or its absence for biotechnological purposes. A *pirC* knockout led to elevated ethanol production under N limiting conditions [16] and absence of *pirC* was also shown to be beneficial for the overproduction of the C storage polymer PHB [17]. Moreover, we have recently shown that the absence of PirC in isoprene producing *Synechocystis* strains led to elevated productivity during N limiting conditions when compared to a control strain with intact PirC [18]. In this work, we sought to increase isoprene production in the model cyanobacterium *Synechocystis* by further optimizing C/N management in an isoprene producing *pirC* deletion strain via prevention of PHB synthesis and enhancement of isoprene synthase activity.

## Methods

### Strains and growth conditions

For subcloning, chemically competent *Escherichia coli* strain DH5αZ1 (Expressys) were used. Cultivation was conducted at 37 °C in LB media in presence of corresponding antibiotics. For cyanobacterial work, a glucose-tolerant *Synechocystis* sp. PCC 6803 was used. *Synechocystis* strains were cultivated in 6-well plates from cryo-stocks in BG11 with respective antibiotics (kanamycin 50 µg mL^− 1^, chloramphenicol 35 µg mL^− 1^,) at 15 µmol photons m^− 2^ s^− 1^ and 30 °C. If not stated differently, cultivation was performed in 100 mL Erlenmeyer flasks containing 20 mL BG11 medium, supplemented with corresponding antibiotics, at 120 rpm, 50 µmol photons m^− 2^ s^− 1^ continuous light at 30 °C.

### Strain generation

For knockout of the *phaAB* genes and overexpression of an additional *ispS* gene copy the previously published phaAB_Eg-IspS/phaAB_CmR plasmids containing either an additional isoprene synthase copy or a sole chloramphenicol resistance cassette [3, 4] were used. As recipient strains the previously published isoprene producing PirC deletion strain and the respective control were used [18]. For *phaAB* deletion, double homologous recombination via natural transformation [19] was performed. In brief, *Synechocystis* cultures were grown to early linear growth phase (OD_750_ = 0.3–0.6), and collected by centrifugation. 1–2 µg of plasmid DNA were added to 200 µL of concentrated cell suspension and incubated for 4–6 h under 50 µmol photons m^− 2^ s^− 1^ illumination and 30 °C. Cells were spread on nitrocellulose membranes (0.45 μm, Cat. No. 10401672, Cytiva, Marlborough, USA) on BG11 agar plates without antibiotics and incubated under standard conditions for 18–24 h, before the filters were transferred to BG11 plates with respective antibiotics (35 µg mL^− 1^ chloramphenicol). Single colonies were streaked onto BG11 plates with antibiotics and evaluated by colony PCR. Positive transformants were cultivated in liquid BG11 with corresponding antibiotics in 6-well plates before cultivation in 100 mL Erlenmeyer flasks, all under standard conditions. Before cryopreservation of the strains, segregation was confirmed via PCR (Figure S1). All strains and plasmids used and generated in this work are described in the supplementary material (Table S1, Table S2, Figure S2).

### Metabolite extraction and quantification

For metabolite extraction cells where rapidly quenched by mixing 1 volume of liquid culture with 2 volumes of precooled (-70 °C) 70% methanol, spun down at 4000 x g, for 5 min. The resulting cell pellets were snap frozen and stored until further use. Next, cell pellets were resuspended in 520 µL of methanol/water mix (2 vol. 70% methanol and 1 vol. MS-grade water, pre-cooled to -20 °C) and mixed with 105 µL precooled (-20 °C) chloroform. Subsequently, 3 freeze thaw cycles of 1 h at -80 °C, de-freezing on ice and subsequent vortexing were performed with subsequent storage at -80 °C overnight. On the next day, samples were defrosted on ice, mixed with 280 µL of pre-cooled MS-grade water and centrifuged at 14 000 x g for 5 min at 4 °C. The upper phase was collected in a fresh tube and the residual lower phase was mixed with 560 µl of pre-cooled MS-grade water, centrifuged at 14 000 x g for 5 min at 4 °C with subsequent collection of the upper phase. This was repeated once more. The resulting samples were split and mixed with 280 µL of pre-cooled MS-grade water before freeze drying overnight.

The dry extracts were then dissolved in 200 µL of MS-grade water and filtered through 0.2 μm filters (Omnifix^®^-F, Braun, Germany). The subsequent liquid chromatography coupled to tandem mass spectrometry (LC-MS/MS) based metabolite quantification has been described before in detail [20]. The compounds were identified and quantified using the multiple reaction monitoring (MRM) values defined in the LC-MS/MS method package and the LabSolutions software package (Shimadzu, Japan). Losses during the extraction procedure were calculated by normalization of signal intensity to that of the internal standard carnitine, i.e., 30 µg of carnitine were added to each sample. Metabolites were determined as relative metabolite abundances, calculated by normalizing signal intensity to that of the internal standard carnitine and their respective OD_750_ mL^− 1^.

### Isoprene measurement

For determining isoprene production, 20 mL of seed culture without antibiotics was transferred to 60 mL air-tight vials (Fisherbrand cat. no. 11540585) and cultivated at 40 °C, 80 µmol photons m^− 2^ s^− 1^ and 150 rpm for 1–15 h [4]. For isoprene quantification, 150 µL of headspace were injected in a gas chromatograph (Clarus 580, Perkin Elmer, Waltham, USA) equipped with a flame-ionization detector and a Porapak QS packed column (Porapak QS 80/80 PE 8000, 1.8 m × 2 mm ID, Cat. No. N9305013-ZW5531, Perkin Elmer). Oven temperature was set to 200 °C for 2.5 min with N_2_ at 20 mL min^− 1^ as carrier gas. Isoprene elution peaked at 1.74 min and was quantified based on the peak area and synthetic standards. OD_750_ was determined after sampling.

## Results

### Abolishment of PHB synthesis in a *pirC* knockout for carbon re-routing

Our previous work showed that, upon N starvation, the absence of PirC can yield higher specific isoprene production activity when compared to control strains with intact *pirC* gene [18]. Nevertheless, the rates achieved were considerably lower when compared to any strain under N replete conditions, with isoprene production ceasing entirely after 3 days of starvation, suggesting that complete removal of N is not applicable for isoprene production [18]. Moreover, several studies have demonstrated that *pirC* deletion causes enhanced synthesis of the C and energy storage polymer PHB due to the inability to accumulate 3-PGA, the metabolic signal required for the induction of gluconeogenesis, when PGAM is dysregulated [9, 17, 18]. Our previous work had shown that PHB synthesis is dispensable for growth and isoprene production of *Synechocystis* [4] whereas the *phaAB* site serves as a reliable insertion site for stable integration into the chromosome. Thus, we set out to engineer an isoprene producing *pirC* deletion strain incapable of PHB synthesis. As parental strains we utilized the previously published *pirC* deletion strain that carries an isoprene production cassette (IC) in the *pirC* locus (strain ΔPirC-IspS), as well as a previously published control strain that carries the same construct in *slr0168*, also coined “neutral site 1” (strain NSI-IspS, Fig. 1B, [18]). In these strains we replaced the *phaAB* operon via homologous recombination with a chloramphenicol resistance cassette yielding strains ΔPirC-IspSΔphaAB and ΔNSI-IspSΔphaAB, respectively (Table S1). Additionally, we recently demonstrated that isoprene synthase (IspS), catalysing the final step of isoprene synthesis, constitutes a major bottleneck for *de novo* isoprene synthesis in *Synechocystis* and introduction of a second gene copy of *Eucalyptus globulus ispS* doubled isoprene production rates [4]. Therefore, we tested whether introducing a stronger C sink in the form of a second isoprene synthase might enhance the effects of dysregulated flux through PGAM towards lower glycolysis. For this, we introduced a second gene copy of *ispS* under control of the constitutively active *Ptrc* promoter into the *phaAB* locus of strains ΔPirC-IspSΔphaAB and ΔNSI-IspSΔphaAB, creating strains ΔPirC-IspSΔphaAB-IspS and ΔNSI-IspSΔphaAB-IspS, respectively (Fig. 1B, Table S1). It should be noted that the latter control strain has, apart from an additional antibiotic resistance cassette, the same genetic setup as the best performing production strain from our previous study (KJ26) [4]. Here we could confirm that, as previously shown, *phaAB* knockout did not impact isoprene productivity in *Synechocystis* during photoautotrophic growth [4], both with and without intact *pirC* (Fig. 1D). Nevertheless, as previously observed, the addition of the second *ispS* copy doubled isoprene productivity in both NSI and Δ*pirC* backgrounds (Fig. 1D) while growth was not affected (Fig. 1C). Being clearly the best performing strains, we henceforth focused on investigating the strains with two isoprene synthase copies, namely ΔPirC-IspSΔphaAB-IspS and ΔNSI-IspSΔphaAB-IspS.

### PirC-PHB deletion causes increased isoprene productivity under fluctuating N supply

**Fig. 2:**
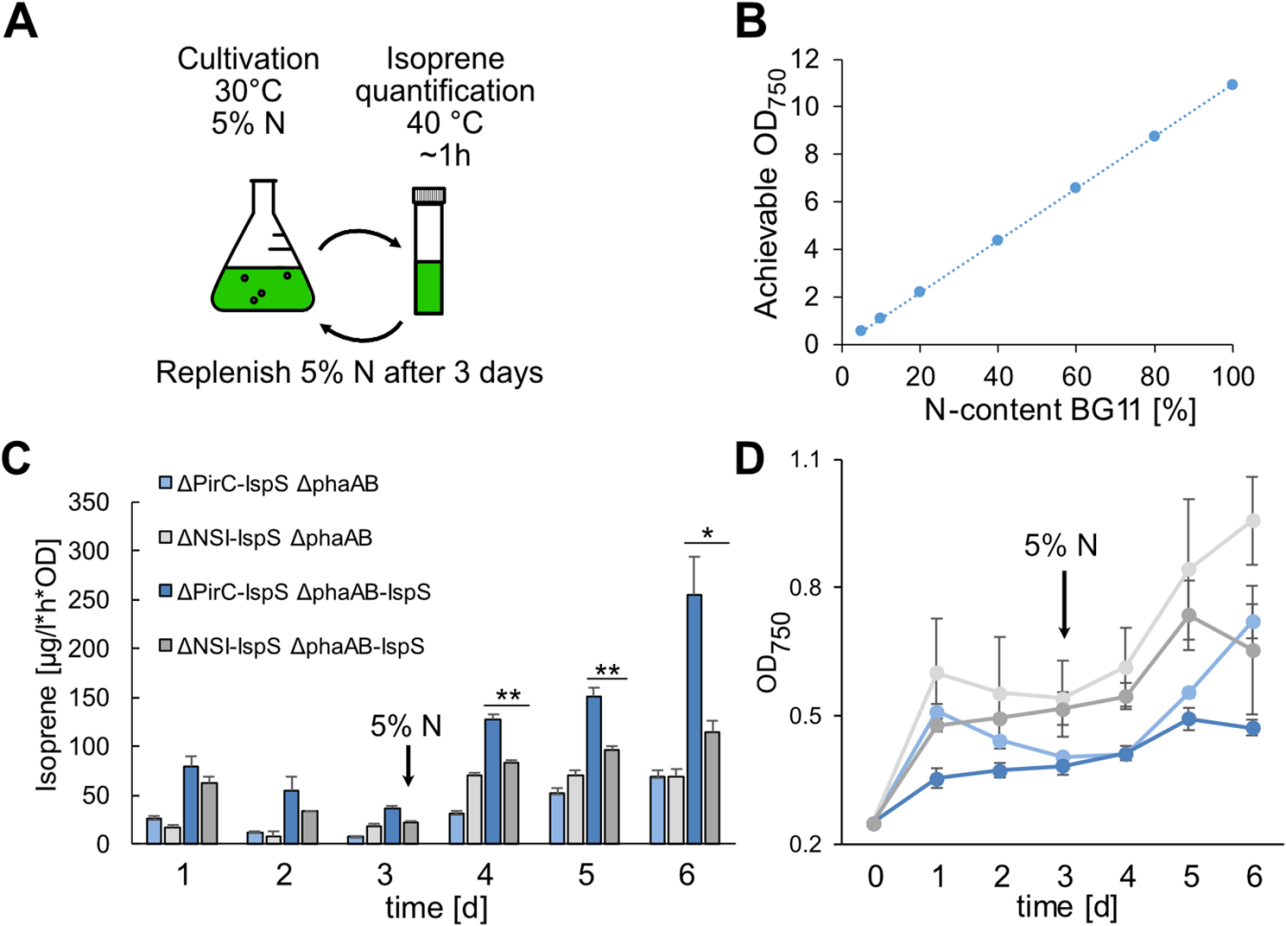
PirC deletion mutant outcompetes control strains under fluctuating N supply. **A** Schematic of N fade out experiment. **B** Calculated values for achievable turbidity (OD_750_) when N is the only limiting factor for growth. **C** Time-course of specific isoprene production activity with 5% N containing BG11, cells were spiked with nitrate, corresponding to 5% of the N content of regular BG11 on day 3. Data shown are average values and standard deviations from 3 independent replicates. significance was tested using paired two samples Student’s *t*-test (ns = *P* > 0.05, * = *P* ≤ 0.05, ** = *P* ≤ 0.01). **D** Growth curves corresponding to isoprene production experiment in (**C**)

In our previous work we showed that isoprene production in the Δ*pirC* strain with strongly reduced N source (5% nitrate with respect to standard BG11 medium) featured similar specific activity as in the NSI background maintained in regular BG11 after 24 h of cultivation. Strict N starvation, however, impaired growth, and as a result isoprene formation, too severely [18]. Thus, we speculated that fluctuating N supply might be a suitable growth mode that enhances C flux towards the MEP pathway when both PHB synthesis and PirC are absent, while not impeding growth too harshly. Moreover, the behaviour of a *pirC* knockout strain in response to N resuscitation has not been reported to our knowledge. To this end, seed cultures of both ΔPirC-IspSΔphaAB-IspS and ΔNSI-IspSΔphaAB-IspS strains were washed twice with BG11_0_ (N-free) and subsequently inoculated at OD_750_ 0.25 in BG11 containing 5% nitrate, followed by daily assessment of growth and isoprene production (Fig. 2). Under the assumption that *Synechocystis* biomass contains 11.3% (g/g) N [21] and an OD_750_ to gCDW/L correlation factor of 0.2, the provided N should suffice for accumulating biomass corresponding to OD_750_ of at least 0.55 before N starvation comes in effect. As expected, increase in cell density halted after the first day, pointing towards N limitation at that phase. After 3 days, sodium nitrate corresponding to 5% N of the standard BG11 concentration was re-added. Upon replenishment with 5% nitrate, cell-specific isoprene titers in the *pirC* deletion background exceeded the ΔNSI-IspSΔphaAB-IspS strain significantly, doubling on day 6 (Fig. 2C). Compared to NSI backgrounds, the Δ*pirC* strains accumulated very little biomass during this phase (Fig. 2D). Thus, N cycling conditions could render a *pirC phaAB* knockout mutant a *bona fide* biocatalyst that primarily invests resources into product synthesis rather than growth, a trait especially desirable for volatile compounds where excessive biomass accumulation is considered futile.

### Absence of PirC and PHB synthesis enhances N use efficiency of photosynthetic isoprene production

**Fig. 3:**
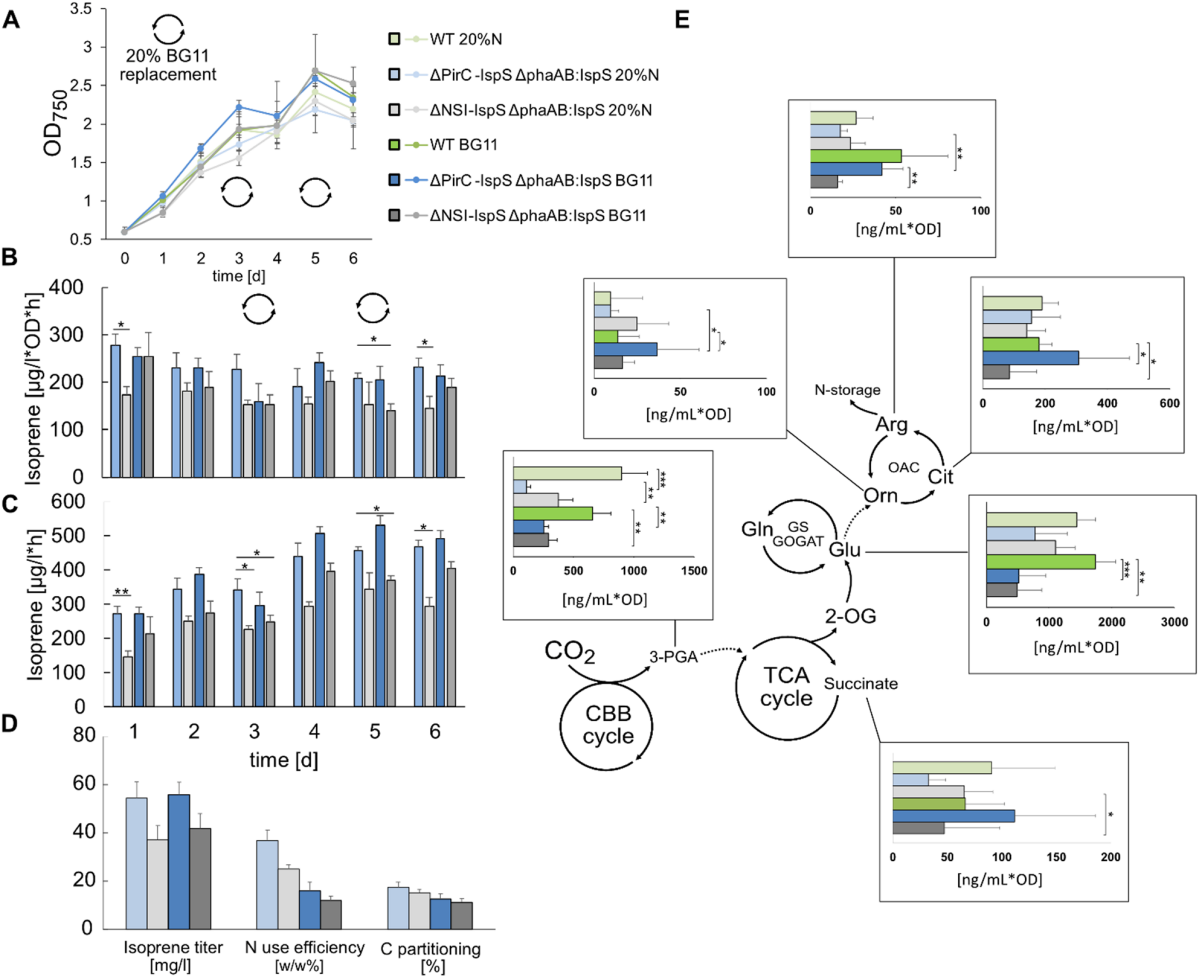
PirC/PHB engineering yields increased N use efficiency for isoprene production. **A** Growth curves of production strains with either 20% or 100% N containing BG11, cells were replenished with 20% fresh BG11 on day 3 and 5. **B** Time-course of specific isoprene production activity. **C** Time-course of volumetric productivity. **D** Cumulative titers, N to isoprene ratio [mg Isoprene/mg Nitrate %] and C partitioning **E**: Metabolite analysis of samples extracted on day 3 of N fluctuation experiment. CBB cycle: Calvin-Benson-Bassham cycle; TCA: Tricarboxylic acid cycle; 2-OG: 2-oxoglutarate; Glu: Glutamate; Gln: Glutamine; GS-GOGAT: Glutamine synthetase/Glutamine-oxoglutarate aminotransferase cycle; Orn: Ornithine; Cit: Citrulline; Arg: Arginine; OAC: Ornithine-ammonia cycle. All data are average and standard deviations of biological triplicates. Significance was tested using paired two samples Student’s *t*-test (ns = *P* > 0.05, * = *P* ≤ 0.05, ** = *P* ≤ 0.01, *** = *P* ≤ 0.001)

Nevertheless, given the large discrepancies in cell densities towards the end of the N fluctuation experiment (Fig. 2D), it cannot be ruled out that the comparatively lower specific activities in the control strains are resulting from light limitation rather than the inability to sustain isoprene formation under N cycling. Moreover, also here, the absolute levels of isoprene formation were low when compared to production experiments under standard, N replete, conditions (Fig. 1D) and only the ΔPirC-IspSΔphaAB-IspS strain was able to reach a similar specific activity on the last day of the experiment, suggesting that 5% N is insufficient to support competitive growth and isoprene formation. Thus, to investigate whether the implemented C flux engineering could indeed be superior compared to hitherto employed strategies, we simultaneously monitored isoprene formation and growth in ΔPirC-IspSΔphaAB-IspS and ΔNSI-IspSΔphaAB-IspS under N replete and 20% N conditions (Fig. 3). For this, seed cultures grown to mid-logarithmic phase were washed twice with N-free BG11_o_ and then inoculated at OD_750_ 0.6 with either regular BG11 or BG11 containing less nitrate, corresponding to 20% of regular BG11 (Fig. 3). Subsequently, these cells were replenished with nutrients after 3 and 5 days by replacing 20% of the culture volume with fresh BG11. Here, it became apparent that the applied N availability was not growth limiting, as evidenced by similar biomass accumulation when compared to WT grown in regular BG11 (Fig. 3A).

Intriguingly, overall isoprene levels were elevated when compared to N cycling at 5%, reaching levels that can be achieved with standard conditions (Figs. 1D and 3B) while ΔNSI-IspSΔphaAB-IspS cultivated with 20% N was outperformed by the other strains/conditions already on day 1 (Fig. 3C). By contrast, specific activity of ΔPirC-IspSΔphaAB-IspS under 20% was comparable to full N supply and remained elevated throughout day 3, when production also under 100% N supply declined in both strains and conditions. After replacing 20% of the cultivation volume of all strains with fresh BG11 medium, production did not recover in the ΔNSI-IspSΔphaAB-IspS strain with 20% N, while it stayed elevated in the ΔPirC-IspSΔphaAB-IspS strain under these conditions. However, even though to a lesser extent, compared to N cycling at 5%, the ΔPirC-IspSΔphaAB-IspS strain with 20% nitrate again accumulated the least amount of biomass among all tested strains and conditions. Thus, it remained questionable if this strain indeed improves isoprene production in the context of process relevant parameters such as volumetric productivity or if the elevated cell-specific activities are an artefact of different light availability and do not translate to an overall improvement of isoprene production over time and space. To this end, we compared the volumetric productivities (Fig. 3C) and estimated the accumulated isoprene titre over time by assuming 24 h of average production within two time points (Fig. 3D). This demonstrated that indeed the PirC-PHB engineering results in elevated space-time yield when compared to ΔNSI-IspSΔphaAB-IspS with regular BG11. To assess C partitioning towards isoprene, we compared the accumulated isoprene with the accumulated biomass assuming 51% C in dry biomass and an OD_750_ to gCDW/L correlation factor of 0.2 (Fig. 3D). This estimation showed that ΔPirC-IspSΔphaAB-IspS at 20% nitrate channelled more fixed C into the product (~ 17%) than the control strain at the same condition (~ 13%) and at 100% N (~ 11%), whereas the *pirC* deletion strain at full nitrogen supply achieved ~ 15% C-partitioning towards isoprene.

Even though isoprene is a hydrocarbon, N is a crucial bulk element for sustaining the surrounding biosynthetic machinery, and strict N limitation was shown to halt isoprene formation entirely [18]. As nitrate constitutes the major cost driver of BG11 with the largest environmental impact [3, 22], we calculated the “N use efficiency”, i.e. the amount of isoprene that was generated with respect to the invested amount of N [w/w%]. Here, the control strain ΔNSI-IspSΔphaAB-IspS with regular BG11 returned around 12%, whereas ΔPirC-IspSΔphaAB-IspS with 20% nitrate achieved over 35% N use efficiency, while still maintaining isoprene production at the same level as the N replete control (Fig. 3D).

To investigate effects by the implemented genetic modifications and N fade out on the abundance of selected intermediates of C and N metabolism, we performed targeted metabolite analysis via liquid chromatography coupled to tandem mass spectrometry (LC/MS-MS) on day 3 (Fig. 3E). 3-PGA, the substrate of PGAM, the enzymatic target of PirC, was significantly reduced in both production strains compared to WT, under 20% nitrate and N-replete conditions. This contrasts previous studies where 3-PGA was elevated under N-replete conditions in a *pirC* null mutant and was only decreased after 48 h of N starvation, when compared to WT [9]. Moreover, ΔPirC-IspSΔphaAB-IspS with 20% N showed significantly reduced 3-PGA levels in comparison to ΔNSI-IspSΔphaAB-IspS. Intriguingly, glutamate, the most abundant amino acid that is produced through ammonium assimilation and used subsequently for many transamination reactions and therefore serves as a pivotal indicator of cellular N status, was also significantly decreased in both production strains under N replete conditions. This effect has not been observed before, neither in a *ΔpirC*-strain with WT background [9] nor in an ethanol production strain carrying a *pirC* deletion [16]. Moreover, the levels of the intermediates of the ornithine arginine citrulline (OAC) cycle, a key pathway for mediating N mobilization and storage in cyanobacteria [23], showed differential levels across different strains and conditions, illustrating the impact of the implemented genetic changes on N metabolism. Specifically, both ornithine and arginine showed significantly reduced levels in ΔPirC-IspSΔphaAB-IspS under 20% nitrate when compared to the same strain at full N supply hinting towards differential N management in this strain.

## Discussion

In this study, we have improved the efficiency of photoautotrophic isoprene production from light and CO_2_ by engineering regulation of primary C metabolism, C sink, and C storage compounds. We achieved an improvement of ~ 30% in isoprene titre in comparison to the most productive *Synechocystis* strains published so far, which served as control strains in this study [4], even when cultivated with significantly reduced N source. As shown before, implementation of a second chromosomally integrated gene copy of isoprene synthase increased the formation rates significantly, illustrating the key bottleneck that is constituted by the final step of isoprene formation [4]. By deletion of both PirC and PHB synthesis, we sought to combine insights from previous studies, re-directing C flux towards isoprene synthesis by fully dysregulating PGAM, while limiting the emergence of C storage compounds. The applied genetic engineering did not impede growth under standard conditions, thus no significant metabolic burden at present formation rates and conditions can be expected. While several studies have employed the *pirC* deletion phenotype by implementing N starvation [16, 17], the behaviour of these strains upon N resuscitation has not been reported. Here we show that, under fluctuating N supply, the *pirC* PHB deletion strains showed growth reduction, while specific isoprene formation rates stayed elevated in these strains. During N limitation, anabolic reactions toward N containing metabolites are tuned down, and growth is arrested. In the WT, the reduction equivalents generated by the light reactions of photosynthesis are then redirected toward glycogen synthesis, whose cellular abundance increases [24]. By contrast, in a *pirC* null mutant, reduction equivalents and newly fixed C cannot be directed in a similar manner towards glycogen synthesis and metabolites of lower glycolysis accumulate [9]. Thus, more precursors should be available for isoprene synthesis via the MEP pathway, if competing pathways such as PHB synthesis are tuned down.

In cyanobacteria, a crucial pathway for coping with fluctuating N availability is the OAC cycle, which serves to build the N storage polymer cyanophycin, a non-ribosomally synthesized peptide consisting of arginine and aspartate [25]. During N and energy rich conditions, OAC directs assimilated N in form of aspartate and glutamine towards arginine eventually leading to cyanophycin build-up, whereas N limitation causes the OAC cycle to rapidly provide N by degradation of arginine [8, 23]. The observed levels of the key OAC metabolites, ornithine, arginine and citrulline, point towards differential activity in the *pirC* deletion strains when N supply is limited. Ornithine, the first intermediate after arginine breakdown, was significantly elevated in the ΔPirC-IspSΔphaAB-IspS strain after 3 days of cultivation in N replete medium when compared to WT. This strain might feature differential N storage management, leading to elevated flux into OAC and consequently cyanophycin build-up, potentially caused by the need to salvage metabolites otherwise channelled into carbon storage compounds.

As previously demonstrated, PirC deletion strains show a phenotype under N limitation that could be deemed as “overflow metabolism”, characterized by the inability to form C storage compounds such as glycogen, leading to excretion of excess C in form of organic acids such as 2-OG and pyruvate [26]. As 2-OG acts as a signal molecule for C/N homeostasis, owed to its role as carbon skeleton for the assimilation of N via the GS-GOGAT cycle [27, 28], the observed differential levels in OAC intermediates could also point towards dysregulated N assimilation in the *pirC* strains. Specifically, excretion of organic acids such as 2-OG might lead to a drop in intracellular 2-OG levels signalling for N rich conditions, thus *pirC* mutants could employ strategies that utilize N abundance by channelling more intermediates into OAC cycle than control strains and WT cultivated under the same conditions. Furthermore, 3-PGA, the substrate of PGAM, was significantly reduced in both isoprene production strains when compared to WT, which is in accordance with previous studies on isoprene producing strains [29]. By contrast, 3-PGA levels were not different in a *pirC* deletion strain with otherwise WT background during N replete conditions and early phases of N starvation [9]. Thus, the observed differences to WT might be due to elevated consumption of 3-PGA in the isoprene production strains. Nevertheless, ΔPirC-IspSΔphaAB-IspS at 20% N showed even more pronounced reduction of 3-PGA when compared to the control strain under these conditions, pointing towards de-repression of PGAM activity.

For any biotechnological process, a key driver of competitiveness is the specific yield, i.e., the amount of achievable product concentration per biocatalyst concentration [30]. For the case of photo-autotrophically produced isoprene, we have demonstrated unprecedented operational stability of the biocatalyst over a large time frame which increases specific yield [4]. Moreover, the specific yield for isoprene is further elevated by the constant release of isoprene from the culture, rendering any in situ product recovery unnecessary while largely increasing the amount of product formed per biomass as the biocatalyst is constantly recycled. Another consideration in the context of scale-up is the accumulation of spent microbial biomass (SMB) whose valorization costs have to be considered to assess the competitiveness of a circular process [31]. Here, we demonstrated that the implemented PirC-PHB engineering allows maintenance of high productivity while restricting biomass formation, which can be tuned by nutrient availability, thus increasing specific yield while reducing SMB.

Limited and fluctuating nutrient supply is prevailing in large scale cultivation systems that run at high cell densities. Moreover, to reduce costs, environmental impact and avoid eutrophication of the environment by elevated nutrient concentrations in the effluent, nutrients should be fed only at demand [32]. Thus, engineering strains that can sustain production when encountering transient nutrient limitation is of great interest. To this end we have demonstrated that applied engineering significantly increases the isoprene output per invested nitrate. As nitrate contributes more than 80% to the total costs of BG11 medium [22] the implemented engineering could reduce operational costs considerably. Moreover, given that reduction of N source to 1/5 did not impede isoprene formation in the control strain, the nitrate content of regular BG11 appears to be excessive for the cell densities and time frames tested here. Further work should assess the effect of other, less cost intensive and more sustainable, organic feedstocks, such a urine [33]. Taken together, the presented engineering strategies could be transferred to a suite of cyanobacterial production processes that are sourced from intermediates of lower glycolysis in order to increase process efficiency.

## Conclusions

Reaching competitiveness of photo-biotechnological *de novo* chemical synthesis requires stark reduction of operational costs. To tackle this, we have engineered production strains with more efficient nutrient usage and carbon partitioning towards the product by targeting central metabolic routes of the host. The presented strategy could be applied to other products derived from lower glycolysis, generally increasing the efficiency of cyanobacterial chassis while reducing operational costs. Taken together, this study demonstrates the effectiveness of regulatory engineering for tuning host metabolism towards over-production and illustrates the necessity for fundamental research on cyanobacterial regulation for extensive metabolic control.

## Supplementary Information

Below is the link to the electronic supplementary material.

Supplementary Material 1.

## Data Availability

All data generated or analyzed during this study are included in this published article and its supplementary information files.

## Abbreviations

2-OG: 2-oxoglutarate
3-PGA: 3-phosphoglycerate
Arg: Arginine
C: Carbon
CBB: Calvin-Benson-Bassham
CDW: Dry cell weight
Cit: Citrulline
CO_2_: Carbon dioxide
DMAPP: Dimethylallyl diphosphate
DXR: 1-deoxy-D-xylulose-5-phosphate reductoisomerase
DXS: 1-deoxy-D-xylulose-5-phosphate synthase
GAP: Glyceraldehyde-3-phosphate
Gln: Glutamine
Glu: Glutamate
GS-GOGAT: Glutamine synthetase/Glutamine-2-oxoglutarate aminotransferase cycle
Idi: Isopentenylpyrophosphate isomerase
IPP: Isopentenyl diphosphate
IspD: 2-C-Methyl-D-erythritol 4-phosphate-cytidylyltransferase
IspE: 4-diphosphocytidyl-2-C-methyl-D-erythritol kinase
IspF: 2-C-methyl-D-erythritol 2,4-cyclodiphosphate synthase
IspG: 4-Hydroxy-3-methyl-but-2-enyl pyrophosphate synthase
IspH: 4-Hydroxy-3-methyl-but-2-enyl pyrophosphate reductase
IspS: Isoprene synthase
MEP: Methylerythritol phosphate
N: Nitrogen
NADPH: Reduced nicotinamide adenine dinucleotide phosphate
NADP^+^: Oxidized nicotinamide adenine dinucleotide phosphate
NSI: Neutral site I (*slr0168*)
OAC: Ornithine-ammonia cycle
OD: Optical density
Orn: Ornithine
PirC: PII-interacting regulator of carbon metabolism
PGAM: Phosphoglycerate mutase
PHB: Polyhydroxybutyrate
Pyr: Pyruvate
TCA: Tricarboxylic acid
