# Supplementary material for: Engineering primary metabolism for sustainable production of isoprene in Synechocystis sp. PCC 6803
Source: Microb Cell Fact. 2026 Jun 5;25:142. doi: 10.1186/s12934-026-03040-8 (PMC13262354; doi:10.1186/s12934-026-03040-8)
Supplement: Supplementary file 1 — Supplementary Material 1. [file 12934_2026_3040_MOESM1_ESM.docx]

# **Supplementary**

**Table S1:** *Synechocystis* PCC6803 strains used and generated in this study

| **Strain Name** | **Genetic modification** | **Reference** |
| --- | --- | --- |
| Wild type | - | (1) |
| ΔNSI-IspS | NS1:*SynIdi-CfDxs-EgIspS* -Km^R^ | (2,3) |
| ΔPirC-IspS | *pirC:SynIdi-CfDxs-EgIspS* -Km^R^ | (4) |
| ΔNSI-IspSΔphaAB | NS1:*SynIdi-CfDxs-EgIspS* -Km^R^ | This study |
|  | *phaAB*:Cm^R^ |  |
| ΔPirC-IspSΔphaAB | *pirC*:*SynIdi-CfDxs-EgIspS* -Km^R^ | This study |
|  | *phaAB*:Cm^R^ |  |
| ΔNSI-IspSΔphaAB-IspS | NS1:*SynIdi-CfDxs-EgIspS* -Km^R^ *phaAB*:*EgIspS*:Cm^R^ | This study |
| ΔPirC-IspSΔphaAB-IspS | *pirC:SynIdi-CfDxs-EgIspS* -Km^R^ *phaAB:EgIspS*:Cm^R^ | This study |

**Table S2** Plasmids used in this study

| **Plasmid ID** | **Plasmid description** | **Antibiotic Resistance** | **Reference** |
| --- | --- | --- | --- |
| phaAB_CmR | pEERM backbone with *phaAB* homology arms for insertion of CmR | CmR | (5) |
| phaAB_Eg-ispS | pEERM backbone with *phaAB* homology arms for insertion of *EgIspS* | CmR | (5) |


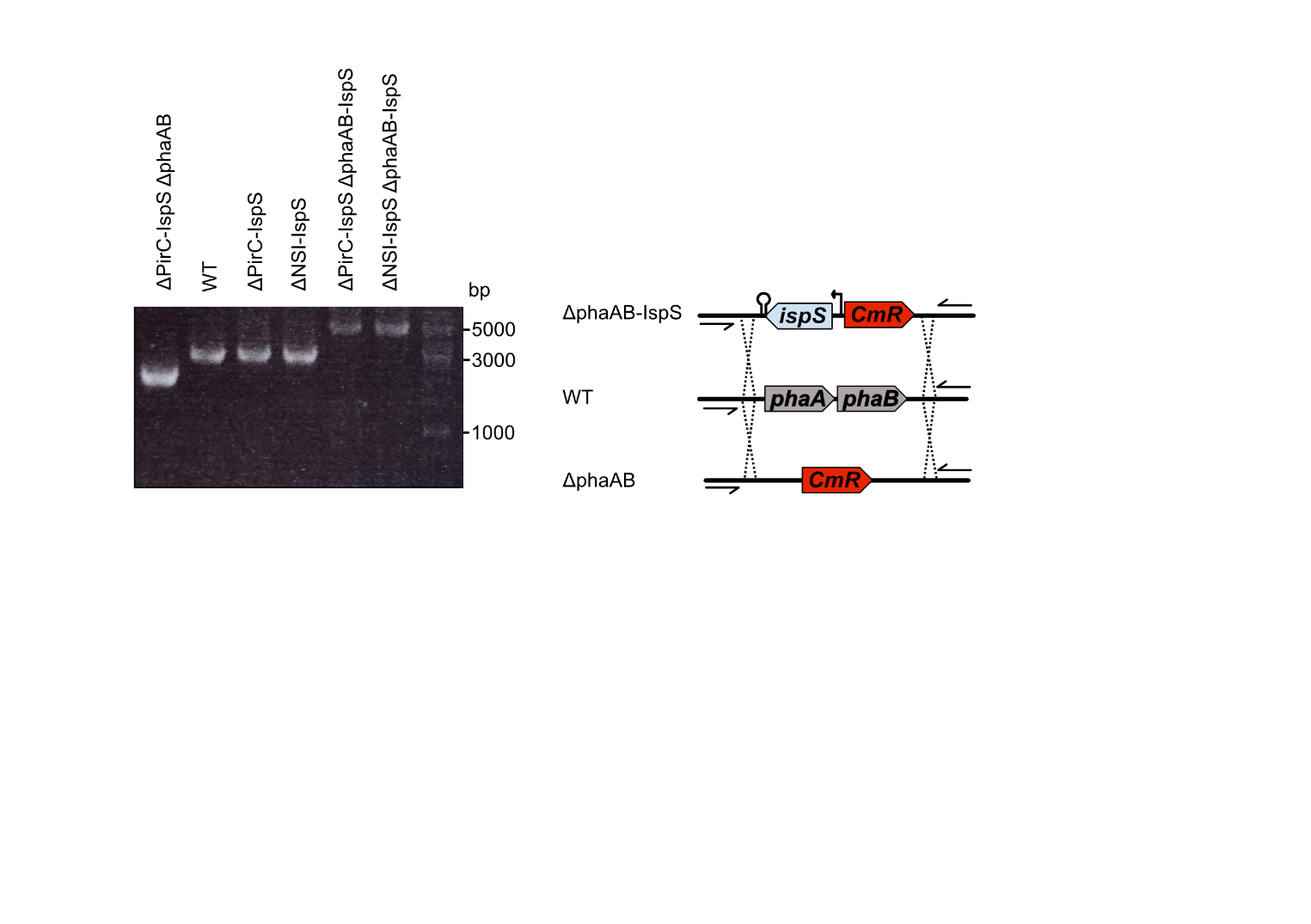


**Figure S1** Genotyping of strains with primers phaAB_KOseg_fw/rev (ggcctagtggctttggacaa/ ttggctccactggcttttttgt). Expected sizes: WT: 3100 bp, *CmR* insertion: 2800 bp, *ispS* insertion: 5000 bp.


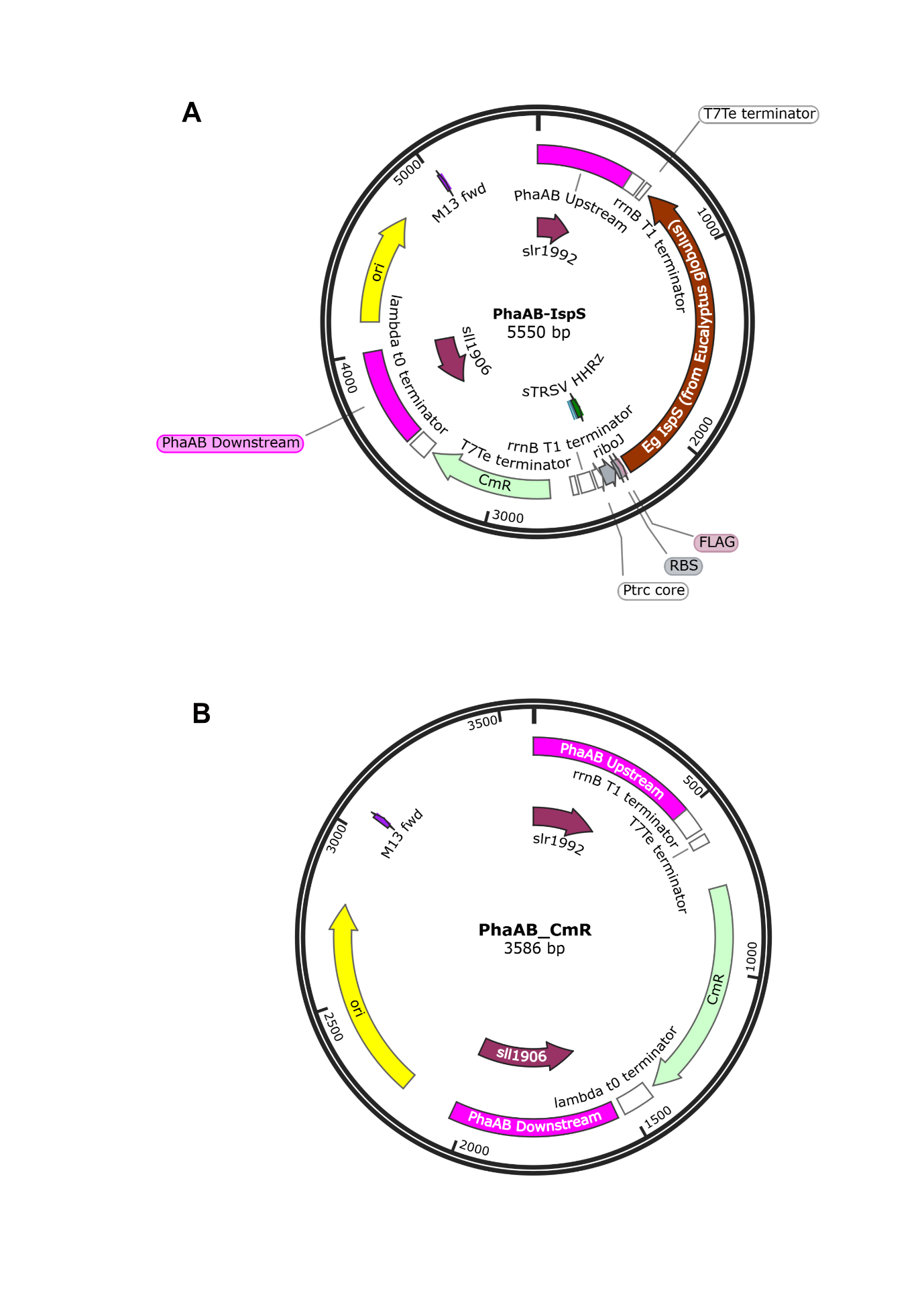


**Figure S2** Maps of plasmids used in this study. A: phaAB_Eg-ispS; B: phaAB_CmR

**Supplemental references**

1. Stanier RY, Kunisawa R, Mandel M, Cohen-Bazire G. Purification and properties of unicellular blue-green algae (order *Chroococcales*). Bacteriol Rev. 1971 Jun;35(2):171–205. doi:10.1128/br.35.2.171-205.1971 PubMed PMID: 4998365; PubMed Central PMCID: PMC378380.

2. Rana A, Gomes LC, Rodrigues JS, Yacout DMM, Arrou-Vignod H, Sjölander J, et al. A combined photobiological–photochemical route to C10 cycloalkane jet fuels from carbon dioxide via isoprene. Green Chemistry. 2022 Dec;24(24):9602–19. doi:10.1039/D2GC03272D

3. Rodrigues JS, Kovács L, Lukeš M, Höper R, Steuer R, Červený J, et al. Characterizing isoprene production in cyanobacteria – Insights into the effects of light, temperature, and isoprene on *Synechocystis* sp. PCC 6803. Bioresource Technology. 2023 Jul;380:129068. doi:10.1016/j.biortech.2023.129068

4. Becker NS, Hufnagel F, Bolay P, Otec K, Orthwein T, Kulik A, et al. Conversion of CO2 into valuable products: Engineering the PirC-PGAM switch in cyanobacteria to direct carbon flux into desired products [Internet]. bioRxiv; 2026 [cited 2026 Feb 17]. p. 2026.02.05.703947. Available from: https://www.biorxiv.org/content/10.64898/2026.02.05.703947v2 doi:10.64898/2026.02.05.703947

5. Janssen KN, Bolay P, Tüllinghoff A, Toepel J, Spindler D, Bühler B, et al. Engineering Cyanobacteria for High-Yield Photosynthetic Isoprene Production With Long-Term Phenotypic Stability. Plant Biotechnology Journal. 2025;n/a(n/a). doi:10.1111/pbi.70395
